# Whole Genome Sequence, Variant Discovery and Annotation in Mapuche-Huilliche Native South Americans

**DOI:** 10.1038/s41598-019-39391-z

**Published:** 2019-02-14

**Authors:** Elena A. Vidal, Tomás C. Moyano, Bernabé I. Bustos, Eduardo Pérez-Palma, Carol Moraga, Eleodoro Riveras, Alejandro Montecinos, Lorena Azócar, Daniela C. Soto, Mabel Vidal, Alex Di Genova, Klaus Puschel, Peter Nürnberg, Stephan Buch, Jochen Hampe, Miguel L. Allende, Verónica Cambiazo, Mauricio González, Christian Hodar, Martín Montecino, Claudia Muñoz-Espinoza, Ariel Orellana, Angélica Reyes-Jara, Dante Travisany, Paula Vizoso, Mauricio Moraga, Susana Eyheramendy, Alejandro Maass, Giancarlo V. De Ferrari, Juan Francisco Miquel, Rodrigo A. Gutiérrez

**Affiliations:** 1 FONDAP Center for Genome Regulation, Santiago, Chile; 2 Departamento de Genética Molecular y Microbiología, Facultad de Ciencias Biológicas, Pontificia Universidad Católica de Chile, Santiago, Chile; 3 Centro de Investigaciones Biomédicas, Facultad de Ciencias Biológicas y Facultad de Medicina, Universidad Andres Bello, Santiago, Chile; 4 Departamento de Gastroenterología, Facultad de Medicina, Pontificia Universidad Católica de Chile, Santiago, Chile; 5 Laboratorio de Bioinformática y Matemática del Genoma (LBMG-Mathomics), Centro de Modelamiento Matemático, Facultad de Ciencias Físicas y Matemáticas, Universidad de Chile, Santiago, Chile; 6 Departamento de Medicina Familiar, Escuela de Medicina, Pontificia Universidad Católica de Chile, Santiago, Chile; 7 Cologne Center for Genomics (CCG), University of Cologne, Cologne, Germany; 8 Medical Department I, University Hospital Dresden, TU Dresden, Germany; 9 Departamento de Biología, Facultad de Ciencias, Universidad de Chile, Santiago, Chile; 10 Laboratorio de Bioinformática y Expresión Génica, Instituto de Nutrición y Tecnología de los Alimentos, Universidad de Chile, Santiago, Chile; 11 Centro de Biotecnología Vegetal, Facultad de Ciencias Biológicas, Universidad Andrés Bello, Santiago, Chile; 12 Instituto de Ciencias Biomédicas, Facultad de Medicina, Universidad de Chile, Santiago, Chile; 13 Departamento de Antropología, Facultad de Ciencias Sociales, Universidad de Chile, Santiago, Chile; 14 Departmento de Estadística, Facultad de Matemáticas, Pontificia Universidad Católica de Chile, Santiago, Chile; 15 Centro de Genómica y Bioinformática, Facultad de Ciencias, Universidad Mayor, Santiago, Chile; 16 Centro de Propagación y Conservación Vegetal (CEPROVEG), Facultad de Ciencias, Universidad Mayor, Santiago, Chile

## Abstract

Whole human genome sequencing initiatives help us understand population history and the basis of genetic diseases. Current data mostly focuses on Old World populations, and the information of the genomic structure of Native Americans, especially those from the Southern Cone is scant. Here we present annotation and variant discovery from high-quality complete genome sequences of a cohort of 11 Mapuche-Huilliche individuals (HUI) from Southern Chile. We found approximately 3.1 × 10^6^ single nucleotide variants (SNVs) per individual and identified 403,383 (6.9%) of novel SNVs events. Analyses of large-scale genomic events detected 680 copy number variants (CNVs) and 4,514 structural variants (SVs), including 398 and 1,910 novel events, respectively. Global ancestry composition of HUI genomes revealed that the cohort represents a sample from a marginally admixed population from the Southern Cone, whose main genetic component derives from Native American ancestors. Additionally, we found that HUI genomes contain variants in genes associated with 5 of the 6 leading causes of noncommunicable diseases in Chile, which may have an impact on the risk of prevalent diseases in Chilean and Amerindian populations. Our data represents a useful resource that can contribute to population-based studies and for the design of early diagnostics or prevention tools for Native and admixed Latin American populations.

## Introduction

Sequencing complete human genomes has greatly expanded the knowledge of our genetic diversity, providing insights into the evolutionary history of man and the bases of human diseases. Large-scale genomic initiatives such as HapMap^1^, the 1000 Genomes Project (1kGP)^2^ or the ExAC initiative^3^ have revealed that individuals from multiple populations carry different profiles of rare and common variants that differ substantially among human continental groups. While current high-coverage full genome efforts have mostly focused on Europeans, Asians and Africans, there is still limited information concerning the genetic structure of Native American groups^4^.

Genome-wide sequence data from ancient and present-day humans has been described for Greenland, Arctic Canada, Alaska, Aleutian Islands and Siberia and used to understand migration pulses into the Arctic regions of America^5,6^. Likewise, whole genome/exome and large-scale genotyping data has been used to study the genetic history, multiple streams of migration and population-genomic variables that underlie patterns of deleterious variation for African, Asian, European, and Native American ancestry in populations of Latin America and the Caribbean^7–10^, as well as the Pacific Northwest^11^. More recently, studies on the demographic history and population structure of admixed South American Latinos has been reported with the aid of genome-wide genotyping technologies^12–14^. However, whole genome sequencing efforts are still scarce, and they should represent a valuable resource for identifying genetic variants influencing susceptibility to develop complex common disorders affecting modern Native American and admixed American populations.

Mapuche-Huilliches descend from early hunter-gatherers who colonized the subcontinent about 15,000 years ago and are the modern representatives of one of the most prominent indigenous groups in the Southern Cone of South America^15^. Here, we sequenced at high-coverage and analyzed the complete genome of 11 individuals belonging to a native Mapuche-Huilliche population from Southern Chile.

## Results

### Overview of Mapuche-Huilliche genomes

Individuals sequenced belong to a community living in Ranco Lake (latitude 40°13′27.62″S, longitude 72°22′50.16″W), in the Los Rios region of Southern Chile (Fig. S1). A common difficulty encountered while studying Native American genetic history is the admixture with individuals from Europe and Africa that occurred since the arrival of Europeans to America in 1,492. For example, Spanish conquerors arrived in Chile in the sixteenth century and began interbreeding with native females, primarily of the Mapuche-Huilliche group, giving birth to the current Chilean population^12,16,17^. Therefore, to ensure *a priori* as little admixture as possible, 11 individuals from the Mapuche-Huilliche community with >3 surnames of Mapuche origin, ABO type O and Rh+, the most common blood type in southern Cone Native Americans, and that were homozygous for a SNV located 13.9 kb upstream of the human lactase gene (*LCT*: C > T-13910; rs4988235), which determines the lactase nonpersistent state in Native Americans^18^, were selected for sequencing (Table 1). This cohort (10 females and 1 male) is hereinafter referred as “HUI”. HUI DNA samples were sequenced using the combinatorial probe-anchor ligation and DNA nanoarray technology of Complete Genomics^19^. We obtained an average of 85% genomic and 98% exonic coverage of at least 30X, with 97% and 98% high confidence calls, respectively.

**Table 1:** Details of HUI individuals selected for this study and genome sequencing statistics.

Assembly ID	Sex	Mapuche surnames	Age at screening	Called genome fraction	Genome coverage 30X	Exome coverage 30X	Mapping yield (Gb)
GS000011194	F	4	22	0.97	0.87	0.98	180.69
GS000011195	F	4	47	0.97	0.87	0.98	181.74
GS000011196	F	4	42	0.97	0.85	0.98	180.88
GS000011198	F	4	46	0.97	0.84	0.98	180.61
GS000011200	F	4	18	0.97	0.84	0.98	177.51
GS000011201	F	4	53	0.96	0.83	0.98	175.46
GS000011215	F	4	43	0.97	0.88	0.98	180.44
GS000012210*	M	4	49	0.96	0.86	0.99	266.52
GS000012242*	F	4	27	0.96	0.92	0.99	357.46
GS000020403	F	4	39	0.97	0.82	0.98	151.00
GS000020711	F	3	60	0.97	0.84	0.98	177.72

Called genome fraction: Fraction of the reference genome with full (diploid) calls in the sequenced sample following assembly; Genome coverage 30X: Fraction of the reference genome bases where coverage is greater than or equal to 30X; Exome coverage 30X: Fraction of the reference exome bases where coverage is greater than or equal to 30X; Mapping yield: Total base-pairs of sequence reads mapped to the reference genome. Samples marked with an asterisk were sequenced twice, thus they have greater mapping yields and average coverage than the other samples.

**Figure 1:**
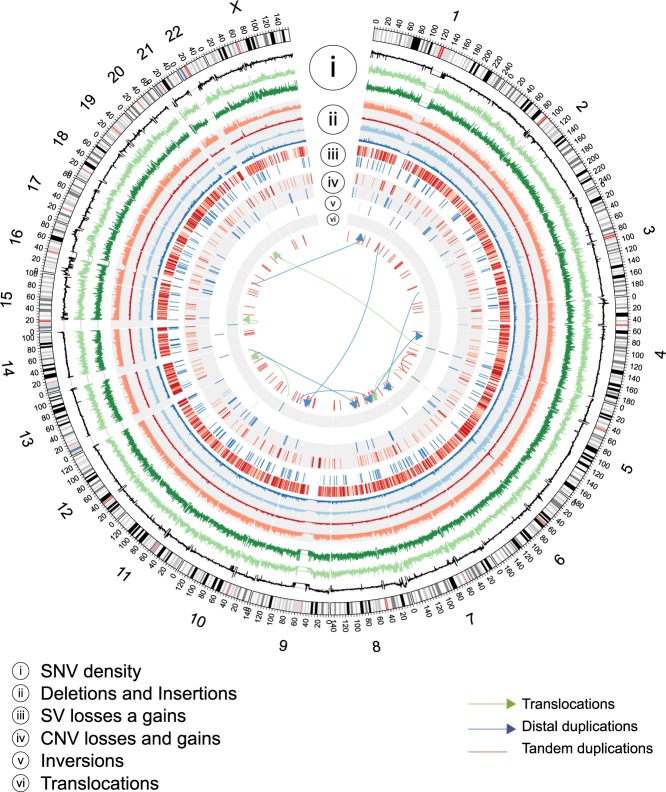
Genetic and structural variants in Mapuche-Huilliche genomes. Circos plot of the spatial distribution of SNV densities (i), deletions and insertions (ii), structural variant (SV) loses and gains (iii), copy number variant (CNV) losses and gains (iv), inversions (v) and translocations (vi). Light or dark colors in different tracks indicate known or novel variants, respectively. Tandem (red lines) and distal duplications (blue arrows) are shown within the inner circle of the plot. Translocation events are shown as green arrows.

Approximately 3.1 × 10^6^ single nucleotide variants (SNVs) were determined for each individual, with a total of 5,847,034 different SNVs in the cohort (Table S1). We found a high level of concordance (99.70%) in the SNV calling rate between genome sequencing and genotyping results obtained using the Illumina Infinium Human Core Exome BeadChip on the same individuals (Table S2). Since the Illumina chip is focused only in exonic and gene-surrounding variants, we determined the genome-wide transition versus transversion mutations (Ts/Tv) ratio for all variants to assess for the presence of false positive calls. We observed that the Ts/Tv ratio in sequenced genomes is 2.1:1 (Table S3), in agreement with the 1kGP expected ratio of 2:1^2^, further confirming the good quality of variants. Moreover, identity by descent (Table S4) and inbreeding (Table S5) analyses indicated that individuals sequenced were not closely related or inbred. A genome-wide summary of main genetic elements in HUI individuals is presented in Fig. 1.

**Figure 2:**
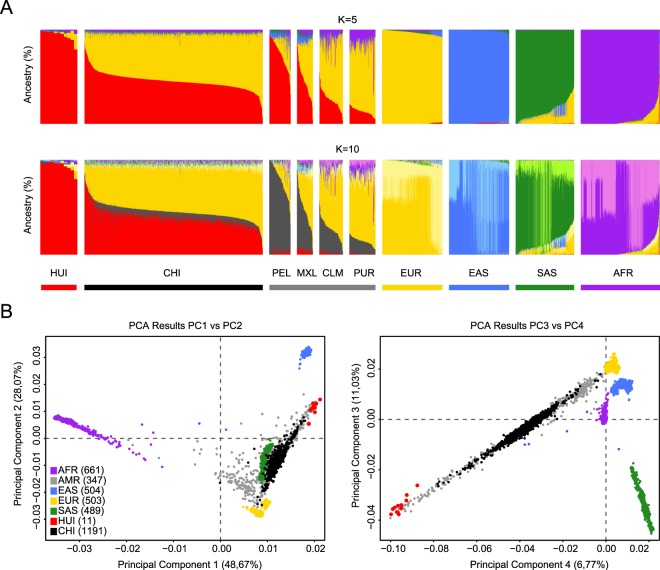
Ancestry analysis of HUI and Chilean Latino individuals. (**A**) ADMIXTURE plots for K = 5 (Continental model) and K = 10 (minimum error model). All 3,706 samples included are depicted as vertical thin bars colored by their corresponding ancestry percentage. HUI genomes are highlighted at the left with thicker bars followed by Chilean Latino genotyped individuals and samples included in 1kGP-phase 3, which are clustered in 5 super-populations (AMR, EUR, EAS, SAS and AFR). For K = 5, the colors were defined as follows: Red for “Amerindian”, yellow for “European”, blue for “East Asian”, green for “South Asian” and purple for “African”. For K = 10, light colors are used to show subcomponents within super-populations EUR, EAS, SAS and AFR. Grey color is used to represent the AMR component common to PEL, MXL, CLM and PUR populations but almost absent in HUI. Bottom thick bars define key colors used in the PCA. (**B**) Principal Component (PC) analysis including the same set of samples (colored dots) and markers. Color legend and number of samples belonging to each super population defined in (**A**) is provided in the legend inside brackets. Left Panel: PC1 vs. PC2, right panel: PC3 vs. PC4. Percentage of variance explained by each component is given in parenthesis in the corresponding axis.

We identified 403,383 (6.9%) novel SNVs that are not included in the dbSNP build 144 release or do not have a reported frequency either in the 1kGP-phase 3 database2, the Exome Sequencing Project^20^ or the Exome Aggregation Consortium (ExAc)^21^ (Table S1). To reduce the amount of variants with missing calls, we applied a calling rate (CR) threshold of 90% giving a total number of 321,803 novel SNVs. From these novel SNVs, most (79.7%, 256,550 SNVs) were present in only 1/22 alleles (Fig. 2A). This trend has been previously reported for other genome sequencing projects, according to the current literature^22^. 175,897 novel SNVs fell in intergenic regions (54.66%), 118,777 in introns (36.91%) and 1,769 in the coding portion of the genome (0.55%, exonic) (Fig. S2). Likewise, we observed 88,173 (19.92%) insertions and 54,718 (12.06%) deletions that are novel and observed in at least 1/22 alleles. In addition, analyses of large-scale genomic events detected 680 copy number variants (CNVs) and 4,514 structural variants (SVs), including 398 and 1,910 novel events (Tables S6 and S7, respectively), that did not overlap any region reported in the CNV map from the Database of Genomic Variants^23^ or the 1kGP-phase 3 release^24^. We found 1,096 genes partially or completely overlapped by ≥1 CNVs or SVs in at least 1/22 alleles (Fig. S3A and Table S8) and 37 genes consistently affected by novel events in all HUI genomes analyzed. Interestingly, some of these large structural events alter the coding sequence of genes and thus may have a potential functional impact (Fig. S3B).

### Global ancestry composition of HUI and Chilean Latino genomes

We used the ADMIXTURE software^25^ to determine the ancestry composition of HUI genomes by comparing a set of 105,252 HUI SNVs that are shared with SNVs present in the complete set of samples of the 1kGP-phase 3 (n = 2,504 individuals)2. In addition, we included a panel of 1,191 Chilean Latinos genotyped by the Affymetrix Axiom World Array LAT 1, to represent the general Chilean population^26^. We ran ADMIXTURE from K = 1 to K = 15 models and obtained near minimum values beyond K = 5 (CV error = 0.51976, Fig. S4A) and a minimum cross-validation error at K = 10 (CV error = 0.51868, Fig. S4B). First, we explored a continental model that considers 5 ancestry components (K = 5), which includes the super-populations from European (EUR), East Asian (EAS), South Asian (SAS), African (AFR) and admixed American (AMR) ancestries from 1kGP-phase 3 data. We found that HUI samples have a high degree of admixed American ancestry (average = 93.8%) with a minimal contribution of other founder populations (Fig. 2A, Top), which validates our ascertainment scheme for selection of individuals to be sequenced. In agreement with recent data regarding the ancestry and complex demographic history of South America^13^, the Chilean Latino panel behaves as an admixed population with a strong genetic contribution of both EUR and AMR ancestries (average = 49.97% and 45.54%, respectively). Other AMR cohorts from 1kGP-phase 3, such as Peruvians from Lima (PEL), Mexicans from Los Angeles (MXL), Colombians from Medellin (CLM) and Puerto Ricans in Puerto Rico (PUR), showed important contributions from EUR and AMR and to a lesser extent from AFR and other super-populations. Second, when we ran ADMIXTURE considering the minimum cross-validation error (K = 10) we observed that all super-populations split into two major components within each cluster, as described for K = 5 (Fig. 2A, Bottom). Notably, we found that a large component of the AMR ancestry in PEL, MXL, CLM and PUR populations (dark gray) is not present in HUI genomes (average = 0.5%) and is marginally represented in Chilean Latino individuals (average = 6.9%, compared with 76.2% in PEL, 42.9% in MXL, 25.6% in CLM and 13.5% in PUR samples). These results suggest that HUI individuals and the broader Chilean cohort derive this genetic component from shared Native American ancestors with low genetic representation in other admixed American populations.

To further explore the genetic structure of the HUI cohort, we used the same set of samples and SNVs and run a principal component analysis (PCA) using EIGENSTRAT^27^ (Fig. 2B). Overall PC1 and PC2 explain 76.7% of the observed variability. PCA results clearly defined world population structure, showing clusters composed of African (AFR), European (EUR) and Asian (EAS and SAS) populations, while the admixed American superpopulation (AMR) was widely distributed between EUR and HUI individuals. Consistent with an absence of recent admixture, the HUI cohort clusters at same axis with the AMR samples (red vs. gray dots, respectively) in the PCA plot. As expected, the largest genetic distance existed between the AFR population and the rest of the groups. In turn, we observed that genotyped Chilean Latino samples (black dots) spread in a relatively narrow cluster that begins at the axis of HUI individuals and ends with individuals from the EUR founder population and overlapped most of the AMR cluster. However, in contrast to admixed Chilean Latinos, the AMR super populations were widely distributed between EUR, AFR and HUI populations. These results are in agreement with the admixture analysis showing that the Chilean Latino population exhibits minimal genetic contribution of other population beyond EUR and HUI/AMR, in accordance to the Chilean demographic history. Unlike other AMR populations with considerable contribution or African/Asiatic immigration (i.e. PUR; average = 15.0% of AFR contribution)^7,28,29^, African and Asian ancestry in Chilean samples was almost negligible (average = 1.5% and 1.1%, AFR and EAS + SAS, respectively).

Analysis of mitochondrial DNA showed that all HUI individuals belong to the Native American haplogroups C and D, two of the major pan-continental founder haplogroups. The majority of genomes sequenced (7 out of 11) belong to the C1b haplogroup and 6 of them were assigned to the clade C1b13 (Fig. S5A), which is a branch found mainly in the Southern Cone of South America between 38° and 42°S^15,30^. While the other 4 individuals belong to the D haplogroup, 3 of them are in the D1g clade, which is found almost exclusively in the central-southern part of Chile and Argentina, and only one is in the D4h3a clade (Fig. S5B), found mainly in the Southern Patagonia^15,30^. These results are in agreement with the admixture data (K = 10, as described before) showing that the genetic component of the HUI cohort differs from the genetic component of other Native American populations living in the northern region of South America.

**Figure 3:**
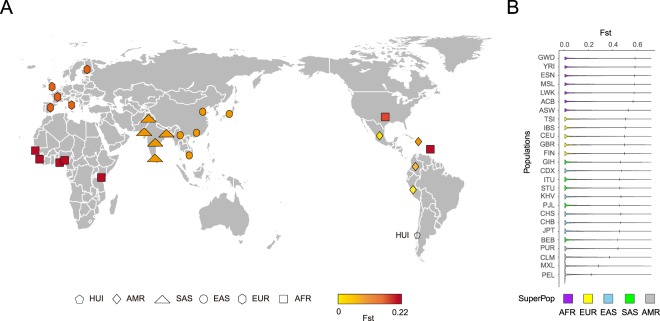
Analysis of the genetic distance (Fst) between HUI cohort and 1kGP-phase 3 population. (**A**) World map showing all 26 populations from 1kGP-phase 3 coming from the 5 super populations (AFR, SAS, EAS, EUR and AMR) and their Weir and Cockerham’s Fst statistic (weighted Fst) from yellow to red according to their genetic distance obtained from the comparison with the HUI sequenced individuals. This figure was created on Adobe Illustrator® CS5 (https://www.adobe.com/) based on a figure made available under the Creative Commons CC0 1.0 Universal Public Domain Dedication (Blank map of the world Equirectangular, https://en.wikipedia.org/wiki/File:BlankMap-World6-Equirectangular.svg) (**B**) Violin plots comparing SNV density between HUI and other 26 populations from 1kGP-phase 3. Fst distributions are sorted by decreasing genetic distance from HUI (top to bottom). Vertical bars on each population plot indicate 95th percentile cutoff. SuperPop = Super populations from 1kGP-phase 3: AFR = Africans, AMR = Admixed Americans, ASN = Asians, EUR = Europeans.

To identify population differentiation between HUI and 1kGP-phase 3^2^, we used pairwise fixation index (Fst) statistics^31^ as a measure of differentiation due to population structure. At the population level, weighted Fst statistics revealed that HUI individuals are genetically closer to admixed American individuals (i.e. PEL, MXL, CLM and PUR), than to individuals from Eastern and Southern Asian or to European and African populations (Fig. 3). These results are in agreement with our admixture results as well as settlement and overall population history of ancient Native Americans^7,10^.

### Variants with Potential Functional Impact in HUI genomes

Genetic variation explaining differential susceptibility to diseases or metabolic conditions derive mostly from studies from old world populations, and more recently from genotyping admixed Latin American populations^12,14,32^. In order to identify functional variants with potential deleterious impact in HUI genomes, we filtered HUI SNVs using ANNOVAR^33^. In brief, we used the Combined Annotation Dependent Depletion (CADD, C score ≥ 20) and FATHMM-MLK (deleterious) databases for predicting the potential functional impact of both coding and non-coding SNVs^34–36^. Variants that passed these filters were termed “Variants with Potential Functional Impact” (VPFIs). VPFIs were then annotated using ClinVar^37^ for their clinical relevance. 520 VPFIs were predicted to be deleterious in several diseases (Table S9). Then, we analyzed overrepresented diseases in the 520 VPFIs using available information on DisGeNET database using WebGestalt^38^. Overrepresented diseases were associated with Kartagener Syndrome, Peripheral Neuropathy, Adverse reaction to drug, Muscular Dystrophy, Gastrointestinal Stromal Tumors, Osteochondrodysplasias, Cardiomyopathy, Dilated, Hypercholesterolemia, Familial, and Neoplastic Syndromes, Hereditary (Table 2). Interestingly, we found that overrepresented diseases in the HUI cohort include 5 of the 6 leading causes of noncommunicable diseases in Chile (diseases of the digestive and cardiovascular systems, musculoskeletal system, sensorial organs, and neoplasms)^14,39,40^.

**Table 2:** Disease enrichment analyses of genes with potential deleterious SNVs.

ID	Name	Hypergeometric enrichment (C, O, E, R)	FDR
umls:C0022521	Kartagener Syndrome	28, 11, 0.89, 12.41	3.26E-07
umls:C0031117	Peripheral Neuropathy	293, 27, 9.27, 2.91	2.55E-04
umls:C0041755	Adverse reaction to drug	54, 9, 1.71, 5.27	9.38E-03
umls:C0339527	Leber Congenital Amaurosis	22, 6, 0.7, 8.62	9.38E-03
umls:C0026850	Muscular Dystrophy	8, 4, 0.25, 15.8	9.38E-03
umls:C0238198	Gastrointestinal Stromal Tumors	8, 4, 0.25, 15.8	9.38E-03
umls:C0029422	Osteochondrodysplasias	15, 5, 0.47, 10.53	9.38E-03
umls:C0007193	Cardiomyopathy, Dilated	47, 8, 1.49, 5.38	1.03E-02
umls:C0020445	Hypercholesterolemia, Familial	16, 5, 0.51, 9.87	1.03E-02
umls:C0027672	Neoplastic Syndromes, Hereditary	48, 8, 1.52, 5.27	1.06E-02

Enriched diseases list identified in the gene set with potential deleterious SNVs in the HUI genomes. The hypergeometric column lists contain: C: number of reference genes in the disease category, O: number of genes enriched, E: the expected number in the category and R: ratio of enrichment. FDR: P-value adjusted by Benjamin-Hochberg multiple test.

For example, Mapuche ancestry has been associated with an increasing mortality due to gallbladder, esophagus and stomach cancer^14^. VPFIs included the rs1801133 variant in the MTHFR gene associated with gastric cancer in Chinese population^41^, the rs1800566 variant in the NQO1 gene, reported as a risk factor in digestive tract cancer in Asian and Caucasian populations^42^ and the rs11887534 variant in the ABCG8 gene, recently associated with gallbladder cancer in Chilean and HUI cohorts^26^. Interestingly, variants in MTHFR and NQO1 genes have not been associated to the Chilean population, however, these variants are highly frequent in HUI and CHI cohorts in comparison with the superpopulations in the 1000 Genomes Project (Table S9). Moreover, we found 7 VPFIs within 5 disease-associated genes in hypercholesterolemia, including *ABCA1*, *APOB*, *LIPC*, EPHX2, and *PON2* genes, two of them directly associated to LDL levels (rs1367117 in APOB)^43^ and to ischemic stroke (rs751141 in EPHX2)^44^. It would be interesting to explore in future studies whether these variants could explain the higher susceptibility to develop hypercholesterolemia reported in Native Americans and admixed Latino populations^45^.

Of particular note are three VPFIs (rs1950902, rs2236225, and rs1801133) in the MTHFD1 and MTHFR genes involved in the folate metabolism pathway. Both enzymes participated in the conversion of homocysteine to methionine and have been implicated in several diseases, including neural tube defects (NTDs), cardiovascular disease, hypertension and various types of cancer^46^. Remarkably, NTD has a high prevalence in Amerindian and Chilean population^47,48^. We also found a VPFIs (rs738409) in the PNPLA3 gene that is strongly associated with alcoholic liver disease^49^, interestingly, Mapuche admixture has been associated with increased mortality due to alcoholic liver disease^14^. In addition, we detected a VPFIs (rs1799999) in PPP1R3A gene and rs200998587 in RBPJL, which are associated with risk of T2D in Amerindian population^50,51^, however, the mortality by diabetes is decreased in Mapuche admixture^14^. Although our results suggest that variants in HUI genomes were associated with diseases, their effects on HUI and Chilean population must be evaluated in further studies.

## Discussion

We herein report the complete genome sequences of 11 unrelated Mapuche-Huilliche individuals and describe common and novel SNVs and large-scale structural variants. Global ancestry composition revealed that HUI genomes sequenced have a minimal contribution of European, East and South Asian and African founder populations (K = 5) and therefore represent an original source of genetic variation for modern admixed individuals living in America. We found that contemporary American populations, including admixed Latinos from Chile and PEL have a high genetic component of an unknown ancestral genetic contribution identified only in HUI genomes. Such contribution decreased in other Amerindian groups represented in MXL, CLM and PUR populations, likely due to the demographic history of Central and North America. Importantly, in these AMR populations we detected a large genetic component that was not present in HUI genomes and that is also marginally represented in Chilean Latino individuals (gray color in K = 10 admixture model), suggesting that AMR ancestors, once they reached and settled in the Southern Cone remained isolated and did not mix with other ancestral groups inhabiting the northern tip of South America until very recently, when Spanish conquerors arrived to America. Such idea is further supported by PCA analyses, mtDNA haplogroups, and Fst analyses, which show that HUI individuals are positioned in a narrow cluster at the edge of the distribution of AMR populations.

Genetically isolated populations are subgroups that often descend from a limited number of founder individuals, share the same environments and may suffer an increased burden of inherited disease^52^. Studies in isolated populations have identified genes and variants associated with Mendelian and complex traits^53,54^. Native Americans from the southern Cone are a group poorly investigated at the whole genome level^4^. VPFIs identified in HUI genome showed allele frequencies were quite different between HUI and 1000G project. Some variants rare or absent in the 1000 Genome project were called by sequencing in HUI cohort. These finding suggest that demographic and adaptive process from HUI cohort could have implication in disease-associated genes observed in HUI and Chilean population.

The complete genome sequence of HUI individuals has several limitations. First, the sample size is too small for being considered as a reference panel and might lead to underestimation of value of allele frequency. However, their admixed Native American background could contribute to understanding the Mapuche-Huilliche genetic component and could become a resource for increasing the sample size. Second, we cannot compare allele frequencies between the HUI cohort and individuals in the 1000 genome project, however, we used the VPFIs identified from HUI discovery cohort and compared qualitatively the allele frequency of HUI and CHI cohorts with the other population. Third, the HUI cohort might not be a representative sample from Mapuche-Huilliche population, however, the identification of HUI individuals not closely related and with a low admixture component with the European populations limited the number of individuals selected. The inclusion of Mapuche-Huilliche from other demographic zones with same inclusion criteria can enrich the whole genome sequencing from Native Americans from the Southern Cone.

We identified multiple VPFIs in functionally related genes that might modulate susceptibility of Mapuche-Huilliche and Chilean population to certain common diseases or disorders. For example, the SNV rs1801133 in MTHFR gene has been correlated with elevated plasma homocysteine^55^. Interestingly, the increase in homocysteine (hyperhomocysteinemia) is associated with cardiovascular, hypertension and neoplasms^46^, being the most common noncommunicable diseases in Chile. The measurement of homocysteine in Mapuche-Huilliche and Chilean populations and the ethnic-specific effect for rs1801133 variant in admixed Chilean with Mapuche-Huilliche ancestry may be interesting factors to evaluate. Future studies aimed specifically to identify genetic susceptibility to these common diseases in populations from the southern cone of America (i.e. GWAS) should take advantage from this novel information by incorporating these variants in discovery phases of case-control or family-based studies.

## Methods

### Ethical considerations

Mapuche-Huilliche individuals from Huapi Island are part of an ongoing longitudinal ultrasonographic study on the prevalence and risk factors of common metabolic diseases in Chile. Informed consent for the study of genetic and metabolic risk factors for prevalent metabolic diseases was obtained from all studied participants in years 1993 and/or 2001^56,57^. Oral and written informed consent from the legal representatives of Huapi Island Mapuche-Huilliche community for whole genome sequencing in some members of the community was obtained in January 2012. The present study was approved by the Institutional review Board for Human Studies of the Faculty of Medicine at Pontificia Universidad Católica de Chile. All methods were performed in accordance with relevant guidelines and regulations.

### Whole-genome sequencing

Mapuche-Huilliche DNA samples were sequenced with the combinatorial probe-anchor ligation sequencing process of Complete Genomics^19^. The standard Complete Genomics bioinformatics pipeline (Assembly Pipeline version 1.10 and CGA Tools 1.4) was used for sequence alignment, read mapping, assembly, and variant call. Human reference genome used was GRCh37.p5 (ftp://ftp-trace.ncbi.nih.gov/1000genomes/ftp/technical/reference/).

### Genotyping of admixed Chilean Latinos

We used a Chilean Latino panel composed of individuals belonging to the ANCORA family health centers located in Santiago-Chile (La Florida and La Pintana) that constitutes an admixed (European-Amerindian) population aged between 20–80 years old from an urban area and representative of the Chilean general population. All subjects were genotyped under the AXIOM® Genome-Wide Platform (version LAT 1) using the GeneTitan® Multi-Channel (MC) Instrument following manufacturer instructions. Samples with discordant sex, elevated missing genotypes rate (≥0.03) or outlying heterozygosity rate (>3 SD) were excluded.

### Identification of total SNVs and Indels

Genomic variants were obtained from MasterVar archives delivered by Complete Genomics (MasterVar file description in http://www.completegenomics.com/documents/DataFileFormats_Standard_Pipeline_2.5.pdf). Only high quality reads were used (low_coverage and half variants were filtered). We registered zygosity for every genomic variation: homozygous variations (both alleles are the same and are different from the reference), heterozygous-reference variations (one of the alleles is different from the reference) and heterozygous-alternative variations (both alleles are different and are different from the reference). Variant Call Format files (VCF) were generated from all 11 MasterVar files using the CGAtools software v.1.8 (http://cgatools.sourceforge.net/). Novel SNVs, Insertions and Deletions were defined as genomic variants that are not included in dbSNP build 144 (http://www.ncbi.nlm.nih.gov/SNP) or that have no frequency reported in the 1kGP-phase 3 Database (http://www.1000genomes.org/home)^2^, the Exome Sequencing Project (http://evs.gs.washington.edu/EVS/), the Exome Aggregation Consortium (http://exac.broadinstitute.org/), and 46 whole-genome sequences from the Complete Genomics public data (http://www.completegenomics.com/public-data/), which were extracted using the ANNOVAR software^33^. Circular representation of SNVs, CNVs and SVs across HUI genomes was drawn with Circos^58^.

### Validation of variants

Variant calling was validated by microarray genotyping using the Illumina Infinium® Human Core Exome BeadChip. The chip consisted in 538,448 variants of which 537,385 are SNVs and 1,063 are indels; 263,929 variants fall in exons. Genotyping study was performed in 9 of the 11 whole-genome sequenced individuals: GS000011194, GS000011195, GS000011196, GS000011198, GS000011200, GS000011201, GS000011215, GS000020403 and GS000020711. The comparison was performed using Variant Call Format (VCF) files which were generated from the Illumina raw genotyping data (Final Report format) taking the genomic positions (chromosome, base pair), the reference allele for the SNV extracted from the NCBI GRCh build 37 reference human genome and the alternative allele from the beadchip annotation data provided by Illumina. The genotype for each SNV (reference homozygote: Hom Ref; reference heterozygote: Het-Ref and alternative homozygote: Hom Alt) was obtained taking the Allele1 and Allele2 Plus information from the raw genotypes. Variants with a Gene Call score (GC) equal or above 0.15 were taken as confident calls, as reported elsewhere^59,60^. The concordance percentage (Conc%) for each individual was obtained taking the number of matching variants and calculating the percentage according to the total number of matching positions.

### Identity by descent (IBD) and inbreeding analysis

The IBD proportion for each pair of the 11 HUI individuals was calculated in PLINK v.1.9, using a subset of 391,284 SNVs that had >90% call-rate and were shared between HUI and 1kGP-phase 3 individuals as described before^61^. Linkage disequilibrium (LD) pruning was applied with PLINK so that no pair of SNPs within a 50 SNPs window present an r^2^ value greater than 0.2. Likewise, inbreeding coefficients were calculated in PLINK v.1.9 for each HUI individual using the same subset of LD-pruned SNVs. IBD and Inbreeding analyses shows little cryptic relationships among the samples used in this work. IBD results show no duplicate individuals of first-degree relationships among our sample (IBD > 0.5). Only two couples of individuals (GS000011194-GS000011201 and GS0000111-GS000012210) show second-degree relationships (IBD 0.25, 0.23, respectively). The inbreeding F coefficient analysis shows only 1 individual above the cutoff of -0.12 (GS000012242, F = −0.14). Negative inbreeding values indicate an excess of heterozygosity or “outbreeding” which could happen because of sample contamination, admixture, or genotyping errors. In this case, this particular HUI sample has the highest European (16.5%) and African ancestries (4,4%) among all 11 individuals. These results indicate HUI individuals sequenced are not closely related or inbred.

### Identification of structural variants

Large-scale structural variants (SVs) were identified by two independent methods: The first one is specifically designed to detect copy number variants (CNVs) based on sequence coverage among samples using a Complete Genomics Hidden Markov Model (HMM) that detects significant abnormal coverage over sliding windows. Under the assumption that a sample is diploid, the method can determine if a genomic segment behaves as a “Gain” or “Loss” in comparison to the reference genome (GRCh37.p5) providing Ploidy -number of times the genomic segment in present- and a PHRED-like score which denotes the confidence of the call (computed as −10*log_10_ of the probability of the assigned call being wrong). When whole genome coverage variability is greater than expected, the sample is assigned to a “no-call” state, which impede CNV analysis. The second method uses the CGATOOLS junctions2events pipeline and is based on junctions analysis – defined as regions of the genome where sequences are not adjacent or in the same orientation as present in the reference genome – that rationalize junctions sets in to the following event types: Deletions, Distal Duplications, Tandem Duplications, Inversions and Translocations. Novel CNVs and SVs events were defined as a variable genomic segment present in at least one sample that did not intersect with any region reported in the inclusive map of the Database of Genomic Variants^23^ or any of the structural variants reported in the latest release of the 1000 genomes project^24^. Genes affected by structural variants were annotated using Refseq genes from the UCSC table browser and classified into the following categories regarding their overlap context with a structural variant: 5-Prime, 3-Prime, Internal, Complete and Chimeric.

### ADMIXTURE and Principal Component Analysis (PCA)

We selected a subset of 105,252 SNVs with common and eligible genotypes within the 2,504 unrelated samples from the 1000 genomes project phase 3 population and the Chilean panel composed of 1,191 samples giving a final total of 3,706 samples to be analyzed. LD pruning was performed with PLINK so that no pair of SNPs within a 50 SNPs window present an r^2^ value greater than 0.2. This dataset was then introduced to the ADMIXTURE^27^ software under default parameters exploring from K = 1 to K = 15 models. Cross validation errors values (CV error) were extracted from each iteration and plotted with R statistical software. Next, this dataset was used to perform a principal component analysis to model ancestry differences between populations using smartpca from EIGENSOFT 5.0 software with default settings^62,63^.

### Maternal ancestry analysis

The complete sequences of mitochondrial DNA of the 11 Mapuche-Huilliches were obtained from the sequencing performed by Complete Genomics. Additionally, 1,016 bp corresponding to the mtDNA control region (rCRS positions 16032–16544 and 051–555) were amplified, purified and Sanger sequenced by Macrogen, South Korea as described^15,30^. Sequences were aligned and edited with Alignment Explorer (MEGA 4.0)^64^. There was complete concordance between Sanger sequencing and mtDNA genome sequences provided by Complete Genomics. Polymorphisms were confirmed directly using Sequencher 4.9 vDemo (http://genecodes.com/). Sequences were grouped by mitochondrial haplogroup and analyses were performed separately. The results were confirmed by comparison with mtDNA tree Build 15 (rCRS-oriented version of Build 15) available on the PhyloTree.org website. Mitochondrial DNA haplogroups from different Native-Chilean and other southern South American populations have been described elsewhere^15,30^. Calculations were performed using the Network 4.5.0 program (www.fluxusengineering.com/sharenet_rn.htm); median joining and maximum parsimony were used as post-processing options.

### Fixation index (Fst) analysis in HUI genomes

Fst analysis was performed using the Weir & Cockerham’s Fst estimator (wcFst)^31^ function inside VCFTools software V.0.1.12b^65^. We obtained Fst statistics at population (weighted Fst) and at SNV level from the comparison between HUI and all individuals from each of the 26 populations of the 1kGP-phase 3: Africa (AFR: YRI, LWK, GWD, MSL, ESN, ASW and ACB), Europe (EUR: CEU, IBS, GBR, FIN and TSI), America (AMR: CLM, PUR, MXL, and PEL), Southern Asia (SAS: PJL, GIH, BEB, STU and ITU) and East Asia (EAS: CHB, JPT, CHS, CDX and KHV). Briefly, to get genotype homogeneity between all HUI individuals, we merged no singleton and no monomorphic SNVs with call rate above 90% with the whole panel of genotypes from 1kGP-phase 3 (also filtered using Vcftools V.0.1.12b) to get a set of common variants between all populations. Violin plots for the population level Fst distributions were done using the ggplot2 R software^66,67^.

### Identification of variants with potential functional impact

Genetic variants were queried for functional impact using ANNOVAR^33^ with the Combined Annotation Dependent Depletion (CADD) database^35^ and FATHMM-MLK (deleterious) databases^35^. Variants that passed these filters were termed “Variants with Potential Functional Impact” (VPFIs). VPFIs were then annotated using ClinVar^37^. VPFIs predicted to be deleterious were analyzed using WebGestalt tools^38^. VPFIs were evaluated against the DisGeNET database for identified overrepresented diseases using the whole genome as a reference set for the hypergeometric test. The disease enrichment were adjusted by the Benjamini-Hochberg method.

## Supplementary information

Supplementary information

Table S9

Table S8

## Data Availability

The data used in this manuscript will be available free of charge upon request for non-for-profit research purposes only. Queries regarding data access should be addressed to R.A.G. (rgutierrez@bio.puc.cl) or J.F.M (jfmiquel@med.puc.cl) or G.V.D. (gdeferrari@unab.cl).
