# Supplementary material for: Whole Genome Sequence, Variant Discovery and Annotation in Mapuche-Huilliche Native South Americans
Source: Sci Rep. 2019 Feb 14;9:2132. doi: 10.1038/s41598-019-39391-z (PMC6376018; doi:10.1038/s41598-019-39391-z)
Supplement: Supplementary file 1 — Supplementary information [file 41598_2019_39391_MOESM1_ESM.pdf]

Supplemental Information

Whole Genome Sequence, Variant Discovery and Annotation in Mapuche-Huilliche Native South Americans

Elena A. Vidal[1,2,15,*] Tomás C. Moyano[1,2,*], Bernabé I. Bustos[1,3,*], Eduardo Pérez-Palma[1,3,*], Carol Moraga[1,2,*], Eleodoro Riveras[1,4*], Alejandro Montecinos[1,2], Lorena Azócar[1,4], Daniela C. Soto[1,2], Mabel Vidal[1,2], Alex Di Genova[1,5], Klaus Puschel[6], Peter Nürnberg[7], Stephan Buch[8], Jochen Hampe[8], Miguel L. Allende[1,9], Verónica Cambiazo[1,10], Mauricio González[1,10], Christian Hodar[1,10], Martín Montecino[1,3], Claudia Muñoz-Espinoza[1,11], Ariel Orellana[1,11], Angélica Reyes-Jara[1,11], Dante Travisany[1,5], Paula Vizoso[1,11], Mauricio Moraga[l2,13], Susana Eyheramendy[14], Alejandro Maass[1,6], Giancarlo V. De Ferrari[1,3,**], Juan Francisco Miquel[1,4,**], Rodrigo A. Gutiérrez[1,2,**]

Correspondence should be addressed to R.A.G. (rgutierrez@bio.puc.cl) or J.F.M. (jfmiquel@med.puc.cl) or G.V.D. (gdeferrari@unab.cl).

Figure S1. Geographic context of HUI samples used in this study. Map of South America depicting the location of Ranco Lake from where HUI samples were obtained**.** This figure using Adobe Illustrator® CS5 (http://www.adobe.com), based on the vector map "South America with Countries - Single Color", obtained from https://freevectormaps.com/.

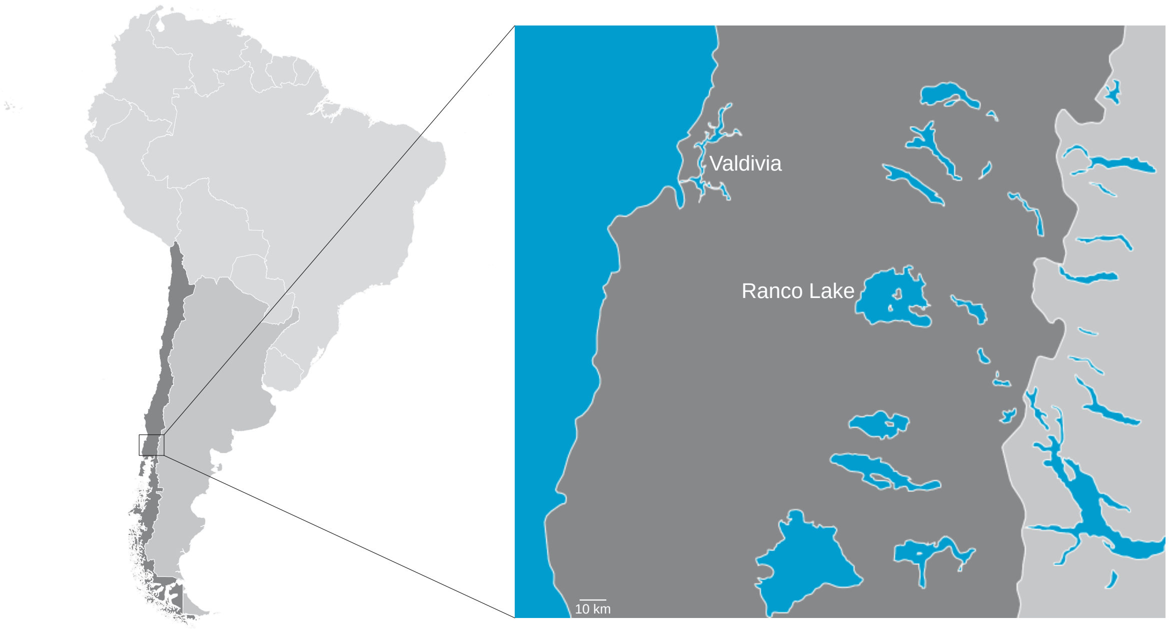

.

Figure S2. Novel SNVs in Mapuche-Huilliche genomes. (A) Allele frequencies for novel SNVs discovered in sequenced genomes. (B) Novel SNVs according to genome location.

A

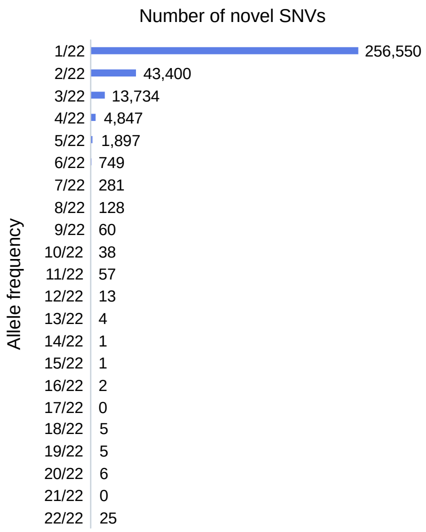

B

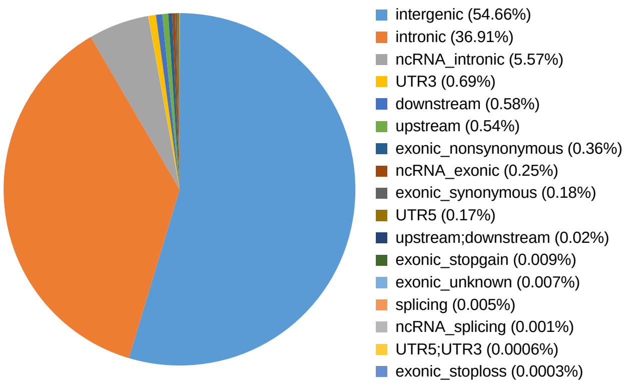

Figure S3. Global map of CNVs in sequenced individuals. (A) Location of 290 CNVs gains (blue) and 390 CNVs losses (red). CNV sites are arranged according to sequenced individuals in tracks. Light or dark colors indicate known or novel variants, respectively. Novel events conserved in all individual analyzed are shown as green triangles. Tracks: 1 (G000011194); 2 (G000011195); 3 (G000011196); 4 (G000011198); 5 (G000011200); 6 (G000011201); 7 (G000011215); and 8 (G000020403); (B) Genomic context and potential functional impact of CNVs shared by all individuals. A thick blue and red bar on top of each gene indicates observed gains and losses, respectively.

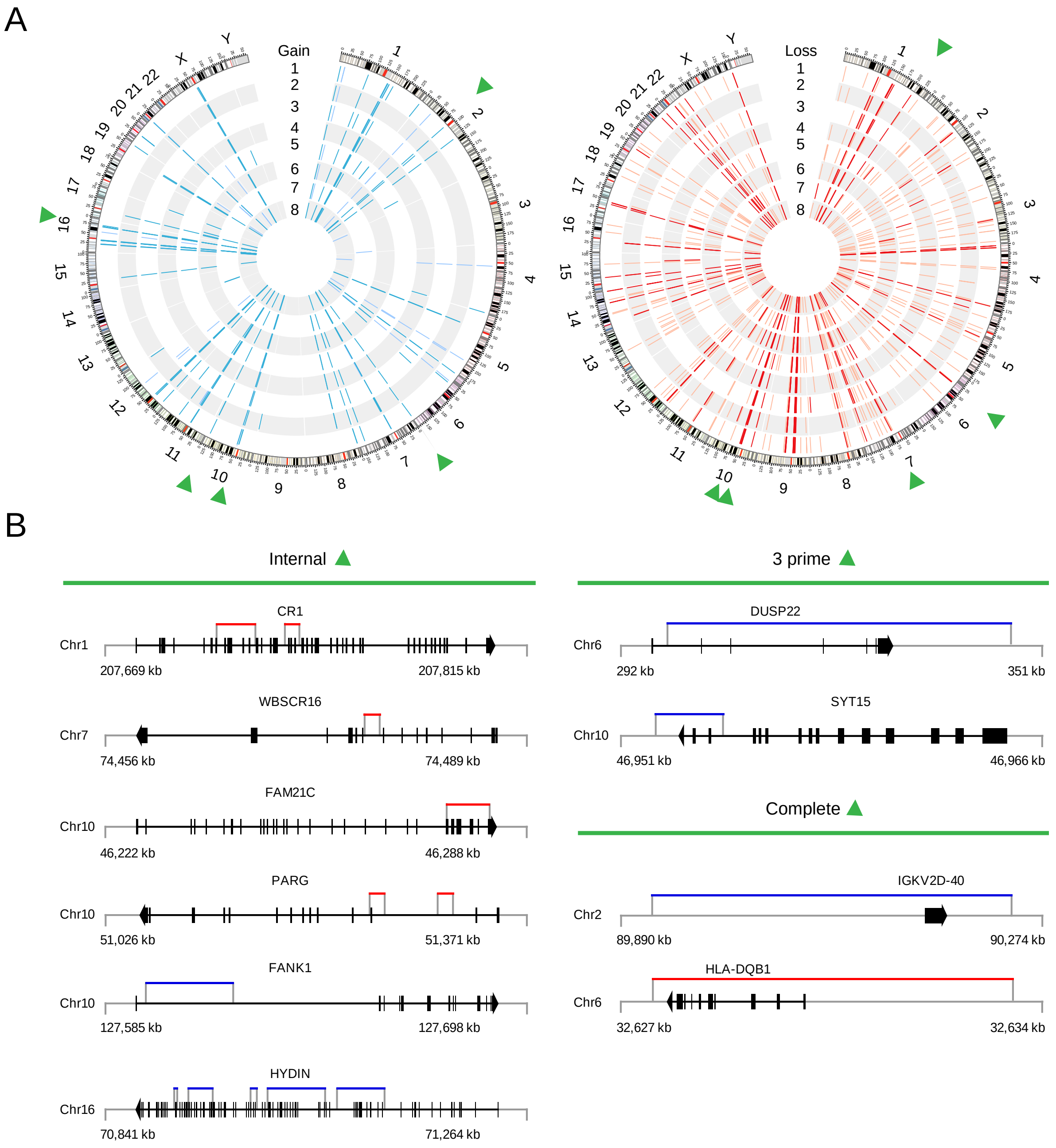

Figure S4. ADMIXTURE analysis of HUI ancestry. (A) Cross-validation (CV) errors are shown for K=1 to K=15 models of ADMIXTURE; (B) Zoom area of (A) with focus on K=5 to K=15 models. Minimum CV error is observed at K=10.

A

B

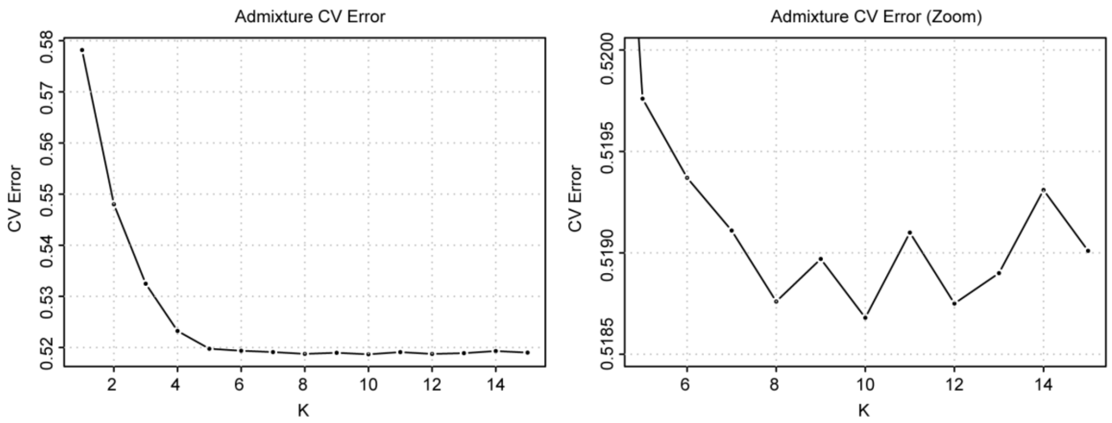

Figure S5. mtDNA haplogroup lineages of Mapuche-Huilliche individuals. (A) Network for the C1 haplogroup. The arrow shows the C1 nodal, characterized by revised Cambridge Reference Sequence (rCRS) differences at 16223, 16298, 16325, 16327, 73, 249d, 263, 290dd, 315+C, 489, 522dd. We also show the southern South American C1b13 haplogroup, characterized by the 258 polymorphism plus C1b core. (B) Network for the mitochondrial D haplogroup. The arrow shows the D1 nodal, characterized by rCRS differences at 16223, 16325, 16362, 73, 263, 315+C, 489. About the D1 haplogroup, the southern South American haplogroup D1g is represented, characterized by the mutation 16187T plus D1 core, and the D4h3a haplogroup, characterized by 16342 and 16241 polymorphisms. HUI individuals from the present study (Huapi-Island) are highlighted in dark blue circles with the respective assembly ID. Haplogroups indicated for each individual were determined from complete mitochondrial sequences.

A

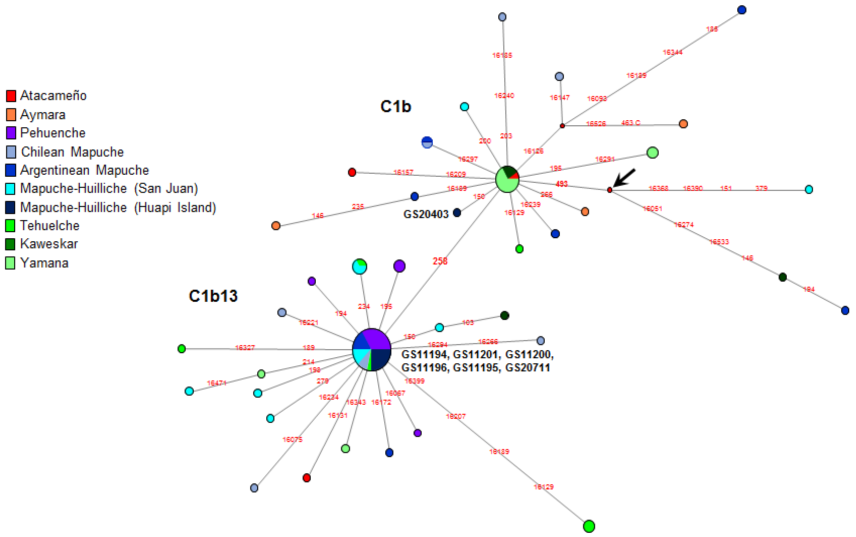

B

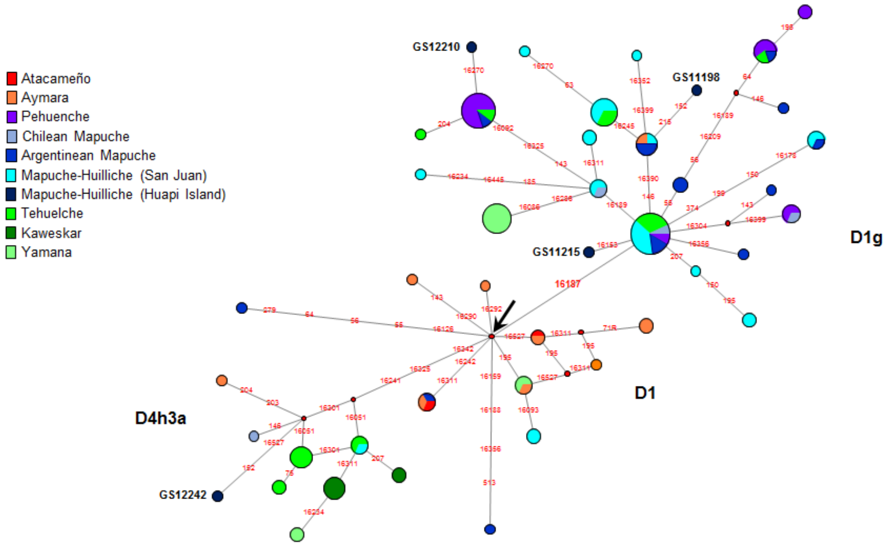

Table S1. Total variants identified in HUI individuals listed by chromosome.

Chr	SNVs	Novel SNVs	MNVs	Novel MNVs	Insertions	Novel insertions	Deletions	Novel deletions
1	445,194	30,238	18,046	9,526	34,697	7,135	35,957	4,355
2	462,032	32,788	19,053	10,377	34,634	6,997	35,359	4,264
3	393,966	28,174	17,287	9,587	28,805	5,536	29,884	3,503
4	422,384	28,848	17,561	9,207	29,285	5,205	31,284	3,257
5	354,460	27,064	14,966	8,410	26,126	4,974	26,884	3,207
6	370,596	24,192	17,430	8,580	27,368	5,217	28,325	3,336
7	338,891	22,801	15,530	8,280	25,440	5,008	25,845	3,046
8	301,338	21,588	12,258	6,666	20,756	4,236	21,420	2,621
9	237,998	16,520	9,719	5,150	17,436	3,517	17,242	2,181
10	289,851	17,280	11,519	5,605	21,799	4,122	22,931	2,651
11	279,792	19,926	13,604	7,655	20,608	4,065	21,206	2,543
12	269,146	19,103	12,360	6,706	20,936	4,072	21,744	2,497
13	212,821	14,028	8,489	4,377	15,859	2,796	16,282	1,590
14	179,874	12,881	7,538	3,993	13,876	2,690	14,100	1,655
15	173,696	10,567	6,897	3,286	13,434	2,636	13,764	1,609
16	180,184	10,960	7,890	3,701	13,037	2,762	12,828	1,577
17	152,629	9,571	6,832	3,245	13,808	3,141	13,738	1,779
18	168,802	10,195	6,324	3,144	12,140	2,138	12,912	1,426
19	130,506	7,699	7,184	3,221	12,346	2,673	12,245	1,766
20	125,336	7,736	5,342	2,589	10,128	2,106	9,852	1,263
21	83,605	5,078	4,147	1,985	6,664	1,166	6,579	697
22	75,040	4,086	3,193	1,419	6,420	1,381	6,350	772
X	196,744	21,790	11,258	7,446	16,699	4,561	16,546	3,090
Y*	2,066	242	183	62	160	39	109	31
Mt	83	28	5	3	1	0	3	2
Total	5,847,034	403,383	254,615	134,220	442,462	88,173	453,389	54,718

SNVs: Single Nucleotide Variants; MNVs: Multinucleotide variants; Asterisk indicates information for chromosome Y for one male individual (GS000012210). Mt: Mitochondrial DNA.

Table S2. Concordance between whole genome HUI sequencing and microarray genotyping.

	Genotyped positions					Matching positions					
**Assembly ID**	**Total**	**Hom ref**	**Het ref**	**Hom alt**	**non matching positions**	**Total**	**Hom ref**	**Het ref**	**Hom alt**	**Genotype Mismatch**	**Conc. %**
GS000011194	515,724	366,412	76,932	72,380	3,257	512,467	364,397	75,842	70,790	1,438	99.72
GS000011195	515,783	367,557	75,272	72,954	3,157	512,626	365,533	74,220	71,397	1,476	99.71
GS000011196	515,881	367,599	75,968	72,314	3,436	512,445	365,430	74,829	70,612	1,574	99.69
GS000011198	515,913	371,069	69,743	75,101	3,475	512,438	368,888	68,663	73,339	1,548	99.70
GS000011200	515,969	367,786	75,012	73,171	3,601	512,368	365,555	73,836	71,469	1,508	99.71
GS000011201	516,102	365,485	80,344	70,273	3,956	512,146	363,091	79,006	68,460	1,589	99.69
GS000011215	516,165	368,012	75,294	72,859	2,696	513,469	366,284	74,373	71,498	1,314	99.74
GS000020403	515,900	366,736	77,729	71,435	3,567	512,333	364,512	76,544	69,690	1,587	99.69
GS000020711	516,112	367,522	76,233	72,357	3,117	512,995	365,689	75,088	70,519	1,699	99.67
Average											99.70

As a validation assay, Mapuche-Huilliche samples were genotyped using the Illumina Infinium® Human Core Exome BeadChip. The number of positions that were genotyped for each individual and the matching positions that were sequenced by Complete Genomics are shown. Hom ref: both alleles are equal to the reference (NCBI GRCh build 37); Het ref: one of the alleles differ from the reference; Hom alt: both alleles are different and differ from the reference; Genotype Mismatch: number of variants that differ between both technologies. Conc. %: percentage of concordance between sequenced and genotyped data.

Table S3. Transition and Transversion mutation ratios in HUI genomes.

SNV	Count
AC	483,880
AG	1,984,115
AT	399,387
CG	510,165
CT	1,985,454
GT	484,019
Ts	3,969,569
Tv	1,877,451
Ts/Tv	2.114

Genome-wide transitions (AG-CT) and transversions (AC-GT-AT-CG) ratios were calculated with VCFtools for a total number of 5,847,020 SNVs as the union of all 11 SNVs in HUI individual genomes.

Table S4. Identity by descent (IBD) analysis in HUI individuals.

ID1	ID2	PI_HAT
GS000011194	GS000011201	0.2547
GS000011196	GS000012210	0.2355
GS000012210	GS000012242	0.1549
GS000011195	GS000011196	0.1361
GS000011195	GS000012210	0.13
GS000011196	GS000012242	0.0943
GS000011198	GS000011200	0.0901
GS000011200	GS000020711	0.0782
GS000011195	GS000012242	0.0728
GS000012242	GS000020403	0.0721
GS000011195	GS000020403	0.0691
GS000011194	GS000011198	0.066
GS000011215	GS000020403	0.0604
GS000011195	GS000011200	0.0565
GS000011196	GS000020403	0.0502
GS000011200	GS000020403	0.0472
GS000011201	GS000020403	0.0469
GS000011200	GS000012242	0.0425
GS000012210	GS000020403	0.0417
GS000011194	GS000012242	0.0415
GS000011200	GS000011215	0.0411
GS000012242	GS000020711	0.041
GS000011215	GS000012242	0.0404
GS000011200	GS000011201	0.0366
GS000011215	GS000012210	0.0286
GS000011194	GS000011200	0.0283
GS000011194	GS000020711	0.025
GS000011194	GS000020403	0.0222
GS000012210	GS000020711	0.0198
GS000011195	GS000011215	0.0197
GS000011201	GS000011215	0.019
GS000011200	GS000012210	0.0187
GS000011196	GS000011215	0.0159
GS000020403	GS000020711	0.0153
GS000011196	GS000020711	0.0106
GS000011196	GS000011200	0.0078
GS000011194	GS000011195	0
GS000011194	GS000011196	0
GS000011194	GS000011215	0
GS000011194	GS000012210	0
GS000011195	GS000011198	0
GS000011195	GS000011201	0
GS000011195	GS000020711	0
GS000011196	GS000011198	0
GS000011196	GS000011201	0
GS000011198	GS000011201	0
GS000011198	GS000011215	0
GS000011198	GS000012210	0

GS000011198	GS000012242	0
GS000011198	GS000020403	0
GS000011198	GS000020711	0
GS000011201	GS000012210	0
GS000011201	GS000012242	0
GS000011201	GS000020711	0
GS000011215	GS000020711	0

IBD analysis was performed in PLINK. ID1 and ID2 indicates the pair of individuals analyzed. PI_HAT corresponds to the proportion of IBD from 0 to 1.

Table S5. Inbreeding (F) analyses in HUI individuals.

ID	O(HOM)	E(HOM)	N(NM)	F
GS000011194	278050	2.85E+05	389701	-0.06461
GS000011195	281054	2.85E+05	389737	-0.03617
GS000011196	279126	2.85E+05	389438	-0.05254
GS000011198	288717	2.85E+05	389369	0.03934
GS000011200	279704	2.85E+05	389286	-0.04611
GS000011201	272696	2.84E+05	388762	-0.1095
GS000011215	280879	2.85E+05	389993	-0.03967
GS000012210	277432	2.83E+05	387773	-0.05641
GS000012242	267725	2.83E+05	387531	-0.1473
GS000020403	274739	2.84E+05	388477	-0.08833
GS000020711	279290	2.84E+05	389076	-0.04848

Inbreeding analysis showing the observed number of homozygotes [O(HOM)], expected number of homozygotes [E(HOM)], number of non-missing genotypes [N(NM)] and the inbreeding coefficient estimate (F).

Table S6. Total number of Copy Number Variants (CNVs) in Mapuche-Huilliche genomes.

	Total CNV	Gain (Novel)	Loss (Novel)
G000011194	253 (139)	95 (73)	158 (66)
G000011195	257 (142)	91 (77)	166 (65)
G000011196	270 (156)	108 (92)	162 (64)
G000011198	282 (162)	112 (91)	170 (71)
G000011200	266 (164)	110 (93)	156 (71)
G000011201	274 (165)	110 (92)	164 (73)
G000011210	ND	ND	ND
G000011215	260 (143)	83 (67)	177 (76)
G000011242	ND	ND	ND
G000020403	248 (136)	84 (68)	164 (68)
G000020711	ND	ND	ND
Total	680 (398)	390 (244)	290 (154)

ND: not determined

Table S7. Total number of Structural Variants (SVs) in Mapuche-Huilliche genomes.

Assembly ID	Total SV (Novel)	Deletions (Novel)	Distal Duplication (Novel)	Tandem Duplication (Novel)	Inversions (Novel)	Translocations (Novel)
G000011194	1,312(506)	1,164(387)	47(47)	93(68)	6(4)	2
G000011195	1,281(492)	1,156(387)	45(45)	76(57)	4(3)	0
G000011196	1,261(506)	1,127(387)	44(44)	85(71)	5(4)	0
G000011198	1,197(460)	1,066(348)	42(42)	81(65)	8(5)	0
G000011200	1,187(455)	1,059(348)	40(40)	83(63)	5(4)	0
G000011201	1,221(479)	1,087(371)	43(42)	84(61)	6(5)	1
G000011210	ND	ND	ND	ND	ND	ND
G000011215	1,331(552)	1,193(432)	43(43)	90(75)	5(2)	0
G000011242	ND	ND	ND	ND	ND	ND
G000020403	1,293(536)	1,186(449)	41(41)	59(41)	6(4)	1 (1)
G000020711	ND	ND	ND	ND	ND	ND
Total (Union)	4,514 (1,910)	3,868(1362)	202(201)	412(329)	28(18)	4(1)

ND: not determined
